# Serum CXCL16 as a Novel Marker of Renal Injury in Type 2 Diabetes Mellitus

**DOI:** 10.1371/journal.pone.0087786

**Published:** 2014-01-29

**Authors:** Leping Zhao, Fan Wu, Leigang Jin, Tingting Lu, Lihui Yang, Xuebo Pan, Chuanfeng Shao, Xiaokun Li, Zhuofeng Lin

**Affiliations:** 1 The affiliated Yueqing Hospital of Wenzhou Medical University, Wenzhou Zhejiang, China; 2 Engineering Research Center of Bioreactor and Pharmaceutical Development, Ministry of Education, Jilin Agricultural University, Changchun, Jilin, China; 3 School of Pharmacy, Wenzhou Medical University, Chashan College Park, Wenzhou Zhejiang, China; University of Louisville, United States of America

## Abstract

**Background:**

Soluble C-X-C chemokine ligand 16 (CXCL16), a scavenger receptor for oxidized low density lipoprotein, has been shown to promote atherogenic effects *in vivo* and to predict long-term mortality in acute coronary syndrome. The aim of this study was to explore the association of circulating CXCL16 levels with diabetic subjects with and without renal disease.

**Methodology/Principal Findings:**

One hundred twenty Chinese subjects, which included patients with type 2 diabetes mellitus (T2DM), diabetic nephropathy (DN), and CKD, as well as healthy controls, were enrolled in this study. Serum CXCL16 levels were examined by immunoassay and other clinical biochemical parameters were tested based on standard methods. Our results indicated that, HDL and LDL cholesterol levels are significantly different in DN but not in T2D patients in comparison with healthy subjects. On the other hand, Serum CXCL16 levels were significantly increased in DN subjects compared with age and gender matched healthy and T2DM subjects (p<0.05 respectively). However, no significant changes in serum CXCL16 levels were found between T2DM and healthy subjects. Furthermore, serum CXCL16 concentration negatively correlated with estimated glomerular filtrate rate, creatinine clearance rate and blood albumin, and positively with 24 h proteinuria, blood urea nitrogen (BUN), creatinine, and uric acid after adjusting for age, gender and BMI in subjects with DN. Multiple stepwise regression analyses indicated that serum CXCL16 levels were independently associated with serum 24 h proteinuria, and BUN (p<0.05 respectively).

**Conclusion:**

Serum CXCL16 may be an indicator of renal injury in subjects with T2DM. Understanding the exact mechanism of elevated CXCL16 in subjects with DN requires further study.

## Introduction

C-X-C chemokine ligand 16 (CXCL16), a member of the scavenger receptors, appears to be the primary receptor for oxidized low-density lipoprotein (oxLDL) based on atherogenesis studies[1]–[5]. It has been shown to promote atherogenic effects *in vivo* through the endocytosis of oxLDL and activation of proinflammatory cascades following ligand binding[1], [4], [6]. For instance, CXCL16 was found to be associated with long-term mortality after adjustment for other risk factors in patients with acute coronary syndrome[7]. Additionally, CXCL16 levels were increased significantly with severity of renal injury caused by hypercholesterolemia and oxLDL generation after unilateral ureteral obstruction[8], [9]. Furthermore, CXCL16 was found to be expressed in podocytes and to act as a scavenger receptor for oxLDL in the pathology of glomerular kidney diseases, notably membranous nephropathy[10]. Recent, our study indicated that serum CXCL16 levels were significantly increased in CKD and gout subjects and were independently associated with a change of renal function[11], [12]. Taken together, these findings implicate that CXCL16 plays an important role in the pathogenesis of renal disease.

Diabetic nephropathy (DN) is a progressive kidney disease and a well-known complication of long-standing diabetes[13]. DN is the most frequent reason for dialysis in many Western countries. Early diagnosis may enable development of specific drugs and early initiation of therapy, thereby postponing or even preventing the need for renal replacement therapy. Our previous work showed that serum CXCL16 levels in DN were significantly higher than those of CKD patients without diabetes[12]. However, whether CXCL16 levels can be attributed to the onset and development of DN from the early stages of diabetes is still unclear. To explore the physiological and pathological characteristics of CXCL16 in subjects with diabetes and DN, we measured the serum CXCL16 levels in 120 Chinese subjects and analyzed its association with a cluster of metabolic parameters. Our data demonstrate that serum CXCL16 levels are significantly increased in subjects with DN, but not diabetes, and they are independently associated with renal function in DN.

## Materials and Methods

### Subjects

A total of 120 Chinese subjects including those with type 2 diabetic mellitus (T2DM, n = 30), diabetic nephropathy (DN, n = 30), chronic kidney disease (CKD, n = 30) and their respective age and sex-matched controls (n = 30) were recruited from the 2^nd^ affiliated Hospital of Wenzhou Medical University. T2DM was diagnosed according to American Diabetic Association criteria (2007), and DN was classified according to either the presence of microalbuminuria (30 to 300 mg albumin/24 hours or an albumin to creatinine ratio [ACR] of 3.4 to 34.0 mg/mmol [30 to 300 mg/g]) or macroalbuminuria (>300 mg albumin/24 hours or ACR >34 mg/mmol [300 mg/g]). DN was also classified on the basis of renal biopsy. Patients with a sustained reduction (≥3 months) in estimated glomerular filtration rate (eGFR) of ≤60 ml min^−1^ 1.73 m^−2^ based on the simplified Modification of Diet in Renal Disease formula were recruited as CKD subjects in the present study. The eGFR values were determined based on our previous report[14]. CKD patients with other complications such as coronary heart disease, diabetes and other relevant diseases would be excluded in the present study. All subjects in the T2DM, DN and CKD groups did not receive any treatment before recruitment. Subjects with following conditions were excluded from this study: biliary obstructive diseases, acute or chronic viral hepatitis, cirrhosis, known hyperthyroidism or hypothyroidism, presence of cancer, current treatment with systemic corticosteroids, and pregnancy. We also included 30 healthy subjects who underwent a routine health examination at the 2nd Affiliated Hospital of Wenzhou Medical College, had no history of medical disease, and were not taking regular medication. All healthy subjects were selected based on the results of a physician's questionnaire and laboratory tests. All studies were approved by the Ethics Committee of Wenzhou Medical College, and all patients provided written informed consent.

### Clinical Data and Laboratory test

All subjects were assessed after overnight fasting for at least 10 hours. Data on demographic characteristics, medical history, current medications, and blood samples were collected from all subjects at the time of enrollment. Blood samples were immediately centrifuged, separated into aliquots, and stored at −80°C for future batched assays. Serum creatinine, phosphate, and albumin were measured with standard commercial assays. Serum CXCL16 (R&D, Minneapolis, MN, USA) and CRP (Antibody and Immunoassay Services, HK)concentrations were measured in duplicate with commercially available enzyme-linked immunosorbent assays according to the manufacturers' instructions in the Core Laboratory of School of Pharmacy, Wenzhou Medical College. All other clinical biochemistry tests were processed in the Clinical Examination Laboratory of the 2nd Affiliated Hospital of Wenzhou Medial College after a single thaw.

### Statistical analysis

All analyses were performed with Statistical Package for Social Sciences version 13.0 (SPSS, Chicago, IL), and the statistical analysis was done similarly as described by Lin Z. et al[15]. Normally distributed data were expressed as mean ± SD. Data that were not normally distributed, as determined using the Kolmogorox-Smirnov test, were logarithmically transformed before analysis and expressed as the median with interquartile range. Student's unpaired t-test was used for comparison between the two groups. Pearson's correlations were used for comparisons between groups when appropriate, and multiple testing was corrected using Bonferroni correction. The variables which correlated significantly with serum CXCL16 (after Bonferroni correction for multiple testing) were selected to enter into stepwise logistic regression. In all statistical tests, P-values <0.05 were considered significant.

## Results

### Characteristics of Study Subjects

Characteristics of T2DM patients (n = 30), DN patients (n = 30), CKD patients (n = 30), and age- and gender-matched healthy subjects (n = 30) are described in Table 1. Compared to the subjects with T2DM, DN patients had higher systolic pressure, fasting insulin, BUN, creatinine, uric acid, phosphate, and 24 h proteinuria compared with T2DM and healthy subjects. They also had lower fasting glucose, 2-h glucose, creatinine clearance rate (CCR), eGFR, blood albumin, and high sensitive C-reactive protein (hs-CRP) levels. Compared to the subjects with DN, CKD disease control subjects had higher total cholesterol, LDL, HDL, CCR, and eGFR levels, and they had lower systolic pressure, diastolic pressure, fasting glucose, 2-h glucose, fasting insulin, BUN, and creatinine levels (Table 1, all p<0.05).

**Table 1:** Anthropometric parameters and biochemical index among subjects with T2DM, DN, CKD and healthy.

Variables	Controls (n = 30)	T2DM (n = 30)	DN (n = 30)	CKD (n = 30)	*p for trend*
Age (years)	47.56±1.60	49.81±2.06	48.65±1.71	49.13±1.89	NS
Gender, male (%)	63%	75%	65%	60.9%	NS
BMI (kg/m^2^)	22.58±0.66	24.07±0.79	22.76±0.500	22.78±0.70	NS
Hypertension					
Systolic pressure (mmHg)	116.0±2.86	120.0±8.94	149.1±3.5^c^	117.7±3.15^†††^	<0.001
Diastolic pressure (mmHg)	74.86±1.85	83.07±2.56^a^	88.55±3.39^c^	78.82±2.16^†^	0.002
The presence of hypertension (%)	0	30%	60%	0	-
Lipid profiles					
Total Cholesterol (mmol/L)	4.82±0.17	5.26±0.30	5.12±0.30	5.83±0.63	NS
HDL-cholesterol (mmol/L)	1.31±0.06	1.24±0.07	1.06±0.09^a^	1.16±0.09	0.032
LDL-cholesterol (mmol/L)	2.64±0.11	2.47±0.24	2.86±0.27^a^	3.68±0.46	0.017
Triglyceride (mmol/L)	1.34±0.15	1.85±0.50	2.17±0.28^a^	1.93±0.34	NS
Glucose metabolism					
Fasting glucose (mmol/L)	4.99±0.11	9.49±0.51^c^	6.10±0.37^c^	4.66±0.13	<0.001
2h glucose (mmol/L)	6.20±0.30	18.39±0.94^c^	11.29±1.49^c^	6.64±0.53	<0.001
Fasting insulin (IU/L)	6.75±0.90	12.36±2.73^c^	15.28±3.62^c^	8.28±2.82	<0.001
Laboratory values					
BUN (mmol/L)	5.16±0.38	5.05±0.32	14.38±2.13^c^	6.97±0.93^††^	<0.001
Creatinine (mg/dL)	62.91±3.798	62.16±3.199	382.4±64.70^c^	154.0±34.25^††^	<0.001
Uric acid (µmol/L)	298.7±13.24	288.7±14.35	432.1±41.95^b^	386.0±23.78	<0.001
CCR (mL/min)	91.49±4.23	98.05±4.48	36.35±7.65^c^	45.76±6.83^††^	<0.001
eGFR	102.0±3.08	108.8±3.97	31.95±7.87^c^	57.30±6.32^††^	0.012
24 h proteinuria (mg/24 h)	52.3±5.71	61.8±12.56	902.7±300.5^c^	723.3±216.5^†^	0.045
24 h proteinuria positive (%)	0	0	27(90%)	23(76%)	-
Albumin (g/L)	41.31±2.08	40.83±0.60	29.96±1.48^c^	25.34±1.78^†^	<0.001
hs-CRP (mg/L)	480.0±130.1	8892±1231^c^	1280.4±188.6^c^	789.7±139.5	<0.001

NS: not significant. Results are expressed as frequencies, mean±SD, or median (interquartile range) as appropriate. ^a^, p<0.05 *vs*. Healthy; ^b^, p<0.01 *vs*. Healthy; ^c^, p<0.001 *vs*. Healthy; ^†^, p<0.05 *vs*. DN; ^††^, p<0.05 *vs*. DN; ^†††^, p<0.05 *vs*. DN.

**Figure 1:**
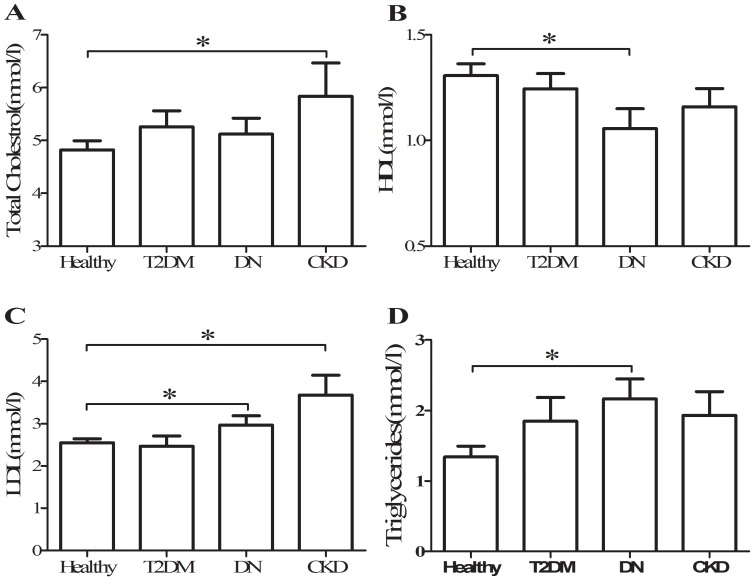
Serum concentration of total cholesterol(A), HDL (B), LDL (C), and triglycerides (D) among T2DM, DN, CKD, and healthy subjects. *, p<0.05.

In lipid profiles, plasma concentrations of total cholesterol were similar in T2DM, DN and CKD patients, and higher than in controls(Figure 1A); whereas HDL cholesterol levels were clearly lower only in DN and CKD than that in healthy but not in T2DM(Figure 1B). LDL cholesterol levels were also higher in DN and CKD than that in T2DM and healthy (Figure 1C). Eventually, triglyceride levels were uniformly higher in T2DM, DN and CKD than that in healthy (Figure 1D). These data suggested that HDL and LDL cholesterol levels are significantly different in DN but not in T2DM patients in comparison with healthy subjects.

### Serum CXCL16 levels are increased in patients with diabetic nephropathy, but not in T2DM

**Figure 2:**
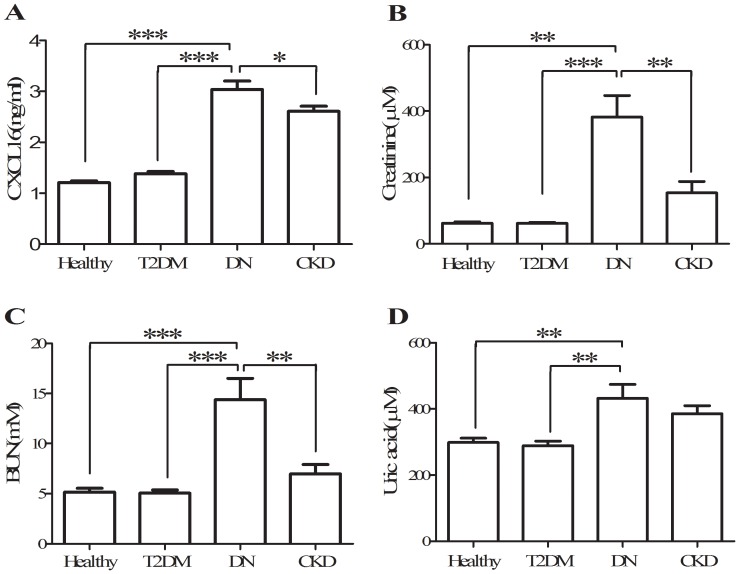
Serum concentration of CXCL16 (A), creatinine (B), BUN (C), and uric acid (D) among T2DM, DN, CKD, and healthy subjects. *, p<0.05; **, p<0.01; ***, p<0.001.

Consistent with our previous reports [11], [12], our data show that fasting serum CXCL16 levels were significantly increased in subjects with CKD (2.65±0.11 ng/ml) compared with healthy subjects (1.30±0.05 ng/ml, p<0.05). Serum CXCL16 levels in subjects with DN (3.04±0.16 ng/ml) were also significantly increased compared with T2DM subjects and healthy controls (p<0.05, Figure 2A). No significant changes in serum CXCL16 levels were observed between T2DM and healthy subjects (1.31±0.03 vs. 1.23±0.04 ng/ml). Additionally, circulating CXCL16 levels were significantly higher in subjects with DN than in age- and gender-matched CKD subjects (p<0.05, Figure 2A), and this is consistent with our previous study [12].

### Serum CXCL16 levels were strongly associated with renal function in subjects with DN

**Figure 3:**
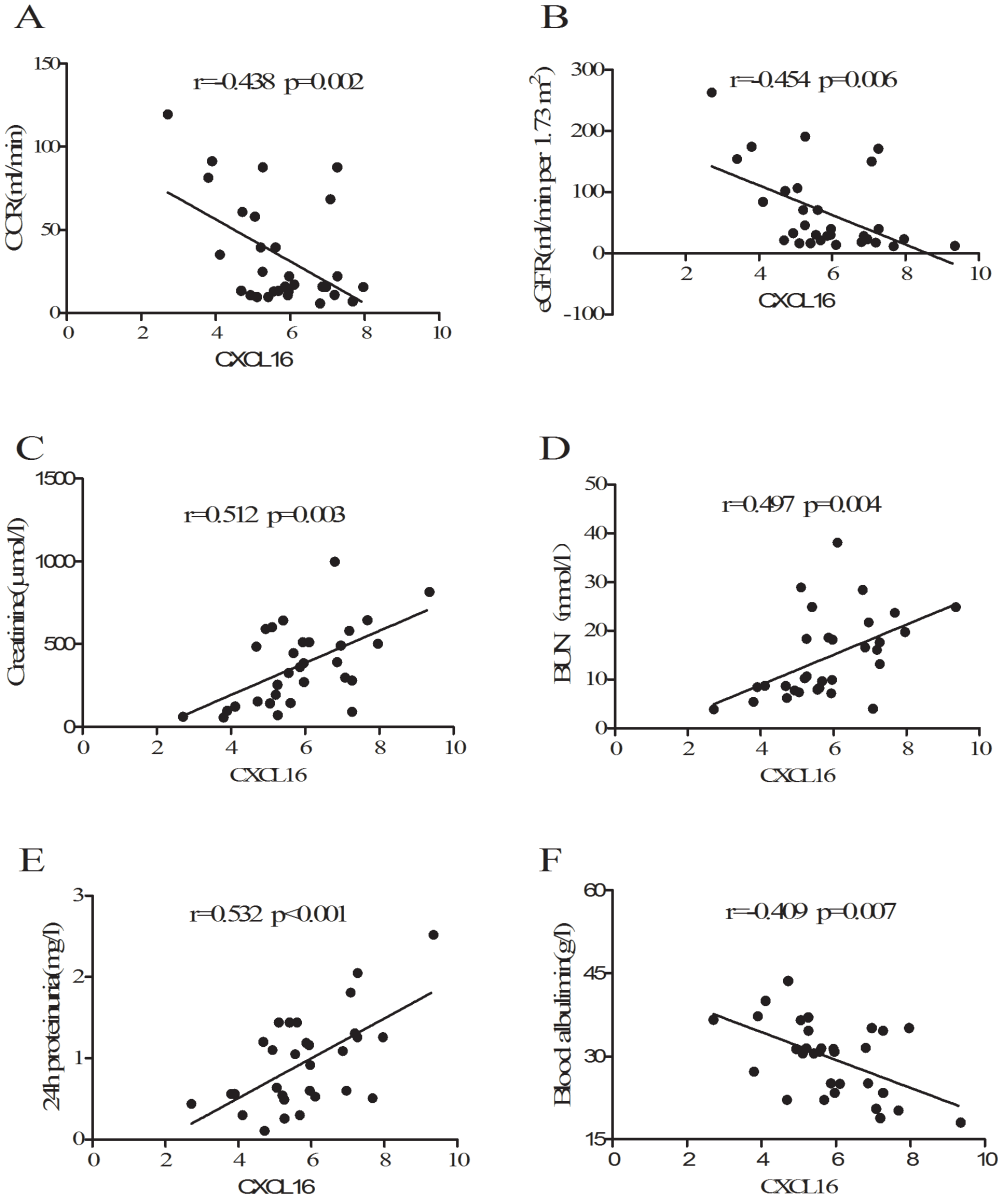
Correlation of serum CXCL16 levels with CCR (A), eGFR (B), creatinine (C), BUN (D), 24 h proteinuria (E), and blood albumin (F) in subjects with DN.

Creatinine, BUN, and uric acid are conventional biomarkers reflecting the decline of renal function in CKD patients [16], [17]. As shown in Figure 2B, 2C, and 2D, serum CXCL16 levels paralleled trends of creatinine, BUN, and uric acid levels among T2DM, DN, and healthy subjects, suggesting that circulating CXCL16 levels may be related to the change of renal function in these patients. To further explore the relationship between CXCL16 and renal function in subjects with DN, correlation analyses were performed. As shown in Table 2 and Figure 3, circulating CXCL16 levels were negatively correlated with endogenous creatinine clearance rate (CCR), eGFR and blood albumin, and they positively correlated with creatinine, BUN, uric acid and 24 h proteinuria in DN subjects after adjustment for age, gender and BMI (p<0.05 respectively).

**Table 2:** Correlation of serum CXCL16 levels with anthropometric parameters, biochemical indexes and other relevant factors in subjects with DN (n = 30).

	Serum CXCL16		Serum CXCL16*	
	r	p	r	p
Age	0.217	NS	-	-
Gender	−0.204	NS	-	-
BMI	0.251	0.042	-	-
Systolic stress	0.362	0.013	0.285	NS
CCR	−0.438	0.002	−0.402	0.013
eGFR	−0.454	0.006	−0.406	0.015
CRP	0.494	0.004	0.298	NS
Blood albumin	−0.409	0.007	−0.331	0.036
24 h proteinuria	0.532	<0.001	0.476	<0.001
BUN	0.497	0.004	0.484	0.008
Creatinine	0.512	0.003	0.445	0.015
Uric acid	0.357	0.011	0.293	0.042

* Adjusted for age, gender, and BMI. NS: not significant.

### Serum CXCL16 levels were independently associated with albumin, BUN and uric acid in subjects with DN

To determine whether serum CXCL16 was independently associated with anthropometric parameters and other relevant factors, multiple stepwise regression analysis involving all the parameters with significant correlations to serum CXCL16 was performed. Our results revealed that serum CXCL16 was independently associated with 24 h proteinuria and BUN after adjustment for age, gender and BMI in all subjects in the present study (P≤0.004, respectively, Table 3). Thus, all other parameters including CCR, eGFR, blood albumin, uric acid and creatinine were all excluded during regression analysis.

**Table 3:** Multiple stepwise regression analysis showing variables independently associated with serum CXCL16 level.

Independent variables	Standardized β	B(95% CI)	t	P
24 h proteinuria	0.588	0.802(0.403 to 1.201)	4.138	<0.001
BUN	0.369	0.047(0.003 to 0.092)	0.311	0.004

## Discussion

Our primary aim in this study was to characterize the clinical manifestation of CXCL16 in conjunction with pathophysiologic measures in diabetes and DN and to explore the relationship between CXCL16 and renal injury in diabetes patients. Our results suggest that CXCL16 is involved in the pathogenesis of renal dysfunction in diabetes patients as supported by two novel findings. First, serum CXCL16 levels were significantly increased in diabetes patients with renal disease when compared with healthy subjects. Furthermore, no significant changes in serum CXCL16 levels were found between healthy and T2DM subjects. Second, HDL and LDL cholesterol levels were significantly different only in subjects with DN and not in T2DM patients without renal injury in comparison with heatlhy subjects. Taken together, these data suggest that CXCL16 may be a novel biomarker involved in the onset and deterioration of renal injury in diabetic patients, and the increased serum CXCL16 levels may be related to the abnormality of cholesterol metabolism in DN subjects.

CXCL16, a chemokine that is mainly expressed on dendritic cells and macrophages, plays an important role in the recruitment of T cells and NK cells[18]–[21]. While much is known from animal-based studies, little is known about CXCL16 in human subjects, particularly in patients with diabetes mellitus. Previous studies have indicated that serum CXCL16 levels are significantly higher in active SLE patients with renal disease than in active SLE patients without renal disease[22]. Furthermore, our previous studies showed that serum CXCL16 levels are significantly increased in subjects with CKD and gout, and these levels are significantly associated with renal function[11], [12]. In the present study, we point to a new pathological manifestation of CXCL16 in the context of diabetic nephropathy. Our data showed that there were no significant changes in serum CXCL16 levels between healthy and T2DM subjects (Figure 1A). However, serum CXCL16 concentrations were significantly increased in DN subjects compared with T2DM and healthy subjects (Figure 1A). These results are consistent with our previous report on CKD subjects with T2DM [12]. This suggests that the elevation of CXCL16 in DN patients may be involved in the pathological progression of kidney disease in T2DM subjects.

Diabetes mellitus (DM) is one of the most prevalent diseases and is associated with increased incidence of structural and functional derangements in the kidneys, eventually leading to end-stage renal disease. Diabetic nephropathy is an important complication in diabetes patients. eGFR, CCR, creatinine, and BUN are conventional biomarkers reflecting changes in renal function in CKD and DN patients [16], [17], [23], [24]. As shown in Table 1, several biomarkers of renal function including creatinine, BUN, uric acid, 24 h proteinuria, eGFR, and CCR in DN patients were significantly increased compared with T2DM subjects without renal disease. We also found that serum CXCL16 concentrations followed changes in a similar manner to creatinine, BUN, and uric acid among the subjects with T2DM, DN and CKD (Figure 1B, C, and D). On the other hand, elevated serum CXC16 levels in subjects with renal damage were already confirmed by our and other studies reports[9]–[12], [25]. In the present study, our data indicated that, elevated serum CXCL16 levels were observed in both DN and CKD patients, even though both of them have different pathogenesis. Take together, these data implied that increased CXCL16 levels in relevant subjects are related to renal damage.

Our previous study indicated that serum CXCL16 levels are significantly increased in subjects with CKD and gout and are independently associated with the change of renal function in these subjects[11], [12]. In the present study, our data indicated that serum CXCL16 levels were strongly associated with eGFR, CCR, creatinine, BUN, and uric acid in DN patients (Table 2, Figure 3), suggesting that elevated CXCL16 levels are closely related to glomerular injury and declining renal function in DN patients. Meanwhile, multiple logistic regression analysis also showed that serum CXCL16 was independently associated with changes in 24 h proteinuria and BUN. Taken together, these results indicate that serum CXCL16 levels are elevated in renal damage patient independently from diabetes. However, the mechanism responsible for the elevation of CXCL16 concentration and its role in the pathophysiology of DN is not fully understood. We speculate that the elevation of CXCL16 expression in subjects with DN may be related to the abnormalites of cholesterol metabolism especially in LDL and HDL. This is supported by the facts that elevation of CXCL16 following with higher levels of oxLDL were found in streptozotocin-induced diabetic mice[26], increased glomerular CXCL16 expression was also accompanied by high levels of oxidized low-density lipoprotein in subjects with glomerular kidney diseases in human[10].

Previous studies indicated that, CXCL16 plays a major role in the uptake of oxidized LDL by podocytes but not by mesangial and tubular renal cells[25]. Furthermore, abnormalities of podocyte structure and function, particularly with regard to LDL metabolism, play a major role in the onset of albuminuria both in diabetic and non diabetic nephropathy[25]. In the present study, we found that the abnormalities of LDL and HDL cholesterol levels were occurred only when overt kidney damage is present, irrespective of glycemic abnormalities in diabetes patients(Table 1 and Figure 2B,C). Take together, these results implied that the LDL-podocyte dysfunction-oxLDL-CXCL16 axis plays an important role in the onset and development of DN. Furthermore, the novel finding of abnormalities of cholesterol metabolism only in diabetic patients with kidney damage may be potentially useful to understand why diabetic nephropathy does occur only in a cohort of diabetic patients.

In summary, this study provides clinical evidence revealing that serum concentrations of CXCL16 are increased in subjects with DN and are independently associated with the loss of renal function. These data imply that CXCL16 may be a novel marker that predicts renal injury in T2DM subjects. There are several limitations in this study. The first one is that the sample size of this study cohort is relatively small. Furthermore, the cross-sectional nature of this study does not allow us to address the causal relationship between CXCL16 and the pathogenesis of DN in patients. Further prospective studies with larger sample sizes are needed to determine whether CXCL16 can be used as a potential biomarker for diagnosing and evaluating the onset and development of DN among DM subjects.
